# Making the connection between health equity and sustainability

**DOI:** 10.3389/fpubh.2023.1226175

**Published:** 2023-09-26

**Authors:** Rachel C. Shelton, Maji Hailemariam, Juliet Iwelunmor

**Affiliations:** ^1^Department of Sociomedical Sciences, Mailman School of Public Health, Columbia University, New York, NY, United States; ^2^C. S. Mott Department of Public Health and Department of OBGYN and Reproductive Biology, Michigan State University, Flint, MI, United States; ^3^Behavioral Science and Health Education, College for Public Health and Social Justice, St. Louis University, St. Louis, MO, United States

**Keywords:** health equity, health inequities, sustainability, implementation science, sustainment, maintenance

## Abstract

Sustainability and health inequities are key challenges in public health and healthcare. Research suggests that only about half of evidence-based interventions (EBIs) are sustained over time, and settings and populations experiencing systemic and structural barriers to health (e.g., poverty, racism, stigma, and discrimination) experience even greater challenges to sustainability. In this article, we argue that an enhanced focus on sustainability in the field of implementation science is critical in order to maximize the long-term health benefits and broader societal impacts of EBIs for all populations and settings. From an equity perspective, a focus on sustainability is particularly critical to prioritize among population sub-groups that have not historically received the benefits of health-related EBIs. We discuss how a health equity framing is essential to sustaining EBIs in under-resourced communities, and requires moving away from a deficit mindset that focuses on why EBIs are challenging to sustain, to one that focuses more on identifying and nurturing existing assets within individuals and communities to increase the likelihood that EBIs are sustained. We conclude with a discussion of future directions as well as recommendations and resources (e.g., frameworks, tools) to advance and make progress toward sustainability from a health equity mindset, including: (1) Actively planning early for sustainability alongside key partners; (2) Tracking progress toward enhancing sustainability and being accountable in doing so equitably for all settings and populations; and (3) Focusing on both equity and engagement early and often throughout the research process and all implementation phases.

## Introduction

Sustainability and health inequities are significant challenges faced in public health and healthcare. Reducing and ultimately eliminating avoidable health inequities will require sustained delivery of programs, practices, policies, products, and treatments that are effective in improving health and reducing health inequities, referred to here as evidence-base interventions or EBIs (1, 2). There have been significant advancements and investments in the development, evaluation, and initial implementation of EBIs that seek to promote health. Yet, what happens to EBIs after initial implementation, especially once implementation support or resources have been removed, has been understudied to date, particularly among lower-resource settings (3). Relatively little is known about the extent to which there is widespread and sustained implementation of EBIs, whether the benefits of EBIs are maintained, and whether there is sufficient capacity built to continue carrying out the EBI as intended (4–8). Thus, despite the promise of EBIs in improving population health (9), many hurdles remain in understanding how to translate these programs to have widespread and long-term impact and benefits outside of well-resourced and controlled settings (1, 10, 11). There are also pressing needs to better understand the return on investment after millions of dollars are spent on initial development and implementation of EBIs, often in settings with limited resources (3).

Despite gaps in understanding, it is well-documented that sustainability is a critical challenge, and that sustainability may be particularly difficult in settings and communities under-represented in research, that face numerous barriers to health, and that have limited access to resources (3, 6, 12). Research suggests that only about half of EBIs are sustained over time (3, 12), and settings and populations experiencing historical and ongoing systemic and structural barriers to health (e.g., poverty, racism, discrimination, and stigma) likely face even greater challenges to sustainability that may be compounded over time (7,13, 14). Lack of sustainability exacerbates health inequities, especially if the discontinuation of EBIs occurs in settings and communities with fewer health-promoting resources (15–18). Thus, failure to sustain EBIs contributes to the maintenance, recurrence, and reinforcement of health inequities. This can result in diminished support and lack of trust of researchers or public health/healthcare systems among communities that have experienced the discontinuation of EBIs following initial implementation (3, 15). Meaningful engagement and amplification of the lived experiences and voices of individuals and communities experiencing inequities is essential to understanding and ultimately overcoming these challenges, and has the potential to enhance both health equity and sustainability (19).

In this paper, as researchers with experience and training in the fields of both health equity and implementation science, we seek to make more explicit the connection between sustainability and health equity. Our definition of health equity here is centered on social justice, where everyone has a fair and just opportunity to be healthy (20); a focus on equity recognizes the injustice of inequities and the underlying root causes that shape them, as well as the community assets and resources needed to address them (11). Additionally, we define sustainability as the extent to which there is continued delivery and ongoing health benefits of EBIs over time, recognizing that EBIs may need to evolve in response to changing contexts to maximize benefits (21, 22). We argue that an enhanced focus on sustainability in implementation science is critical to maximizing the health and societal impact and benefits of EBIs for all populations and settings, particularly among those that have not historically received the benefits of EBIs. Additionally, we highlight future gaps and opportunities, as well as recommendations and resources (e.g., frameworks and tools) to advance and make progress toward sustainability from a health equity perspective.

## Why sustainability matters for health equity

There are many reasons why sustaining EBIs matters for health equity and why researchers and funders must prioritize sustainability in order to be more accountable in making progress toward eliminating inequities. *First*, because health equity research is under-resourced and has not historically been valued as a priority for all researchers or funders, most EBIs were not developed, evaluated or implemented in populations or settings under-represented in research (2). Under-represented populations experience persistent health and social inequities that limit efforts to improve health for all groups. Thus, there has been a major disconnect between the EBIs that researchers are typically seeking to implement and ultimately sustain and their long-term fit in addressing the real-world needs and priorities of underserved communities. Prioritizing the sustainability of equity-focused programs and policies will help prevent avoidable suffering and care for those who are unwell, while creating lasting conditions that promote health from the beginning, in which all can truly thrive. *Second*, of the EBIs implemented, there is often a delay or latency period for many health-related interventions, where the impact or benefits to the community or at the population level may not be seen until many years after initial implementation (3, 6). Therefore, discontinuing programs results in suboptimal public health benefits, particularly among the populations and settings that would benefit from them the most. These include organizational settings, communities, and populations that have fewer social and economic resources or face structural barriers to health. Additionally, discontinuing programs prematurely will mean not only failing to achieve the health impacts, but also not seeing the gains of investments in health in other broader economic, social, and policy changes that are typically only observable over time [e.g., across many years (23)].

*Third,* discontinued programs can reflect a substantial loss of investment in valuable time and resources for initial implementation on the part of funders, organizations, leadership, practitioners, and administrators. This may result in frustration and wariness about future implementation efforts, constituting a major challenge particularly among settings with limited resources and many competing demands (e.g., low-income communities, neighborhoods, and groups experiencing the harrms of structural racism) (7, 13). *Finally*, abandoning, abruptly stopping, or failing to continue delivery of EBIs may also bring disillusionment to service users and community members, and reinforce negative perceptions and distrust or mistrust of research and health services among community partners and the broader public, with subsequent implications for future engagement of communities (7, 24).

## Challenges and considerations in promoting sustainability and health equity

Sustainability is intricately linked with health equity, as unsustainable or discontinued EBIs can lead to disparate health outcomes across settings and population sub-groups (5, 13). There is a need to go beyond traditional definitions of sustainability, to expand the construct to include more diverse voices and perspectives to advance understanding of what is needed to maximize the long-term delivery and benefits of EBIs among under-resourced communities. For any implementation effort, including but not limited to sustainability, a focus on health equity should center and uplift community values (25–27). Efforts to sustain EBIs should take into consideration the transformative nature of community engagement and its strong potential to lead toward social justice, particularly when involving the redistribution of power, resources, and decision-making (28, 29). Such an approach is particularly relevant for low-resource and historically marginalized communities where health and economic inequities are evident along the lines of race and socioeconomic position. As has long been recognized (26, 28), meaningful community engagement and partnership are central and foundational to sustainability efforts and have the potential to reduce clinical and public health inequities and improve population health (26).

At the heart of any effort to sustain EBIs from an equity mindset are the key partners who are engaged in or impacted by interventions and implementation efforts. Such partners are critical to engage to understand the long-term use, needed resources, and ongoing improvements and adaptations of EBIs over time (27). Successfully sustaining EBIs focused on reducing health inequities requires engaging a range of key partners throughout the planning, implementation, and adaptation process to increase the fit between EBIs and local context/resources, while also addressing dynamic and emerging issues that might impede sustainability (14, 16, 22). Establishing processes to facilitate ongoing and meaningful engagement with key partners in the setting where EBIs are deployed is essential to managing and supporting the sustainability of an EBI within a changing context. This was the case in a study on sustaining community based participatory research (CBPR) efforts in three urban research centers in Detroit, New York, and Seattle. Israel et al. (30) found that lack of time and resources, alongside maintaining the commitment of partners over time, were key challenges identified that impacted participation in EBIs. However, having the “right people at the table” (including program champions and local partners) while ensuring and communicating clear program benefits to all partners, were essential to overcoming these challenges and sustaining community engagement efforts over time. *Key partnerships and establishing processes to facilitate ongoing, meaningful engagement can support the coordinated actions needed to improve health equity and sustainability*.

Nonetheless, many EBIs were not developed or evaluated with equity in mind (2). In most cases, meaningful involvement of communities experiencing inequities as partners in the design and implementation process is also limited, further diminishing the potential to center community values with the goal of social justice and representation of racially and socioeconomically diverse communities and settings (1, 2). Many EBIs and implementation efforts have also not considered the extent to which structural determinants like systemic racism shape not only health inequities but also intensify inequities in implementation reach, uptake, delivery, and long-term sustainability (29, 31). Additionally, many of the EBIs prioritized for delivery do not typically focus on creating changes at the policy or systems level that might have more sustainable impact (32). Given this disconnect in the nature of the evidence base, implementation science as a field is not always well poised to maximize progress toward health equity or sustainability. In many cases, the EBI being delivered is not a good fit from the start (e.g., was not developed with/for the community, is not culturally or contextually appropriate, is complex and costly, is not acceptable or feasible in light of limited resources and time, or does not align with existing organizational context or readiness), which will have critical implications for its long-term delivery and health impact (13). The appropriateness of an EBI in any setting will require not only an understanding of readiness for change, but also knowledge of the presence of competing initiatives, acute human resource challenges, and organizational support and alignment for the EBI (6). A deeper understanding of the fit between the EBIs and the context in which it is implemented is crucial for reducing health inequities and informing strategies to enhance sustainability. Lack of attention to fit and organizational readiness may result in programs and strategies that are not sustained or offer minimal benefits to address inequities. Tools like the Hexagon Tool (33) and the Organizational Readiness for Implementing Change (34) assessment may be useful to understand both fit and organizational readiness to deliver an EBI in a specific context. *To prioritize equity as an essential component of sustainability, it is important to assess the fit between context and the intervention, and consider making adaptations to the context or the intervention to align with key priorities and existing resources in the setting.*

More research on fit and the context in which EBIs are implemented is necessary for understanding how EBIs and strategies should evolve and adapt to promote sustainability. Practitioners and implementers may find that sustaining the core components of EBIs with high fidelity is challenging, particularly in settings that have limited resources. It is increasingly recognized that some adaptations to EBIs may actually be helpful and necessary in delivering and ultimately sustaining EBIs (35). Such adaptations may be useful in order to enhance fit within specific settings and organizational contexts or to reflect the sociocultural characteristics of communities that differ from the original setting or population in which the EBI was developed (36). Not making such adaptations may exacerbate health inequities, particularly if EBIs are not adapted to address social determinants of health (e.g., lack of transportation) and align with new sociocultural contexts (35). Inattention to adaptation may result in lower reach and engagement of the EBI for communities that face structural barriers to health (e.g., if an EBI is not adapted to reflect patient literacy levels, financial barriers, and language in a rural clinic that serves predominately Spanish-speaking Latino populations) (1). *To make progress toward equity, it is critical to track and empirically evaluate the types of adaptations that matter for enhancing sustainability of EBIs, including in low-resource settings and populations experiencing inequities, while still identifying and maintaining those core components that are essential for achieving health outcomes.*

Additionally, research suggests that specific contextual factors may be important to consider when seeking to sustain interventions in settings experiencing resource and health inequities; for example, empirical research suggests that partnerships, organizational capacity, resources, program burden, fit with context, and staff attrition are key determinants of sustainability in low-resource communities (13, 14). Additional factors may be relevant in global settings; for example, Iwelunmor et al. (7) reviewed 41 studies across 26 countries in sub-Saharan Africa and found that community mobilization, engagement, and resources were essential to consider, as well as working with existing resources, providing adaptable interventions that are flexible to local context, and considering the broader societal and political context and upheavals. There may also be different learning needs, literacy and educational levels, and language preferences of the populations being served, varying perceptions of EBI burdens and benefits, as well as trauma, harm, and distrust of public health/medicine based on experiences of racism in communities (13, 14). Thus, in lower-resource settings, as EBIs and strategies are selected, it is important to understand key contextual determinants in those settings that may impact sustainability, including differences in patient populations as well as organizational infrastructure and resources available. Existing sustainability frameworks can provide a useful starting place for conducting such contextual assessments [e.g., (15, 37)], but may require refinements to address specific contexts and health equity considerations.

It may also be useful to specifically understand equity considerations and map assets within communities and settings early on to help facilitate local ownership and enhance sustainability. Such efforts may help ensure that delivery of and refinements to EBIs reflect local cultural norms, system realities, and challenges (e.g., healthcare worker shortages), and the broader socio-political context. Frameworks like the PEN-3 cultural model (38–40) with its focus not only on barriers, but also on factors within settings that are positive and existential, may help to uncover and amplify assets critical for sustaining EBIs and advancing health equity in communities under-represented in research. *Contextual assessments can be useful in planning for and promoting sustainability with a focus on health equity (40, 41); this will require that we not only approach barriers to sustainability from a deficit perspective only* (i.e.*, limited resources or what society is doing poorly*)*, but also from an asset mindset, including existing resources that can be tapped to foster sustainability among systemically marginalized groups and settings, and opportunities to further enhance existing capacity in a more sustainable way.*

## Discussion

Here, to help chart a path forward for the field of implementation science to synergize, enhance impact, and advance the science, we highlight key recommendations and additional considerations to advance sustainability from a health equity mindset.

### Recommendations to advance a focus on equity and sustainability in implementation science

#### Actively prioritize and plan early for sustainability alongside key partners

To effectively apply implementation science to promote health equity and build trust with community partners, it is essential to actively plan for the sustained and equitable delivery and impact of EBIs in a dynamic way over time. This will help researchers be more accountable in tracking the extent to which continued EBI delivery and implementation over time reduces or exacerbates health inequities. This requires that we explicitly monitor and track the extent to which program activities are delivered and sustained equitably across all settings and population sub-groups. Planning and tracking progress allows the possibility of intervening early to identify and address challenges to implementation and sustainability as they arise across implementation phases. An extension of the RE-AIM framework (Reach; Effectiveness; Adoption; Implementation; Maintenance) was introduced to enhance and promote sustainability, with a focus on dynamic context and health equity over time (42). This may be a useful tool to guide tracking of where and when inequities are reduced or exacerbated across implementation phases and what needs to be adapted or refined to promote long-term sustainability equitably. Specifically, this extension recommends: (1) consideration of dynamic, longer-term sustainability across the life cycle of EBIs (at least 1 year post-implementation and on an ongoing basis); (2) iterative or periodic application of RE-AIM assessments to guide possible adaptations needed to plan for and enhance long-term sustainability; and (3) explicit consideration of equity and cost as cross-cutting issues that have implications for sustainability and should be assessed and ideally addressed across all RE-AIM dimensions (42).

Additionally, developed in the context of ongoing research among young people in Nigeria, Iwelunmor et al. (19) introduced PLAN (or how People Learn, Adapt and Nurture the core values of an intervention), which may enable the engagement of partners, as well as the planning and development of more practical and realistic strategies that foster sustainability. Practitioners, end users, and policymakers typically do not engage with or learn about the science of sustainability or how to enhance sustainability efforts through the peer-reviewed literature (19). To help ensure that lived experiences connect with and informs scientific research and that research findings are translated and reach local practitioners, it may be useful to incorporate and apply PLAN to help understand and communicate when, where, how, and why sustainability matters for a particular EBI from the perspective of local community members and what dissemination channels are appropriate to reach a range of key audiences (19). Such planning may help to identify the right people who matter early for sustaining EBIs equitably and foster learning across the life-cycle of EBIs (including strategies to improve the fit of EBIs in practice). Additionally, initiating planning processes may nurture existing assets within settings that may facilitate ownership and long-term support of and capacity to deliver EBIs after initial implementation.

#### Monitor progress of efforts to enhance sustainability and track the extent to which sustainability is equitable across a range of settings and population sub-groups

As researchers are building the empirical evidence base around the impact of implementation and sustainability strategies (43), it is critical to also track and build an evidence base around the extent to which whether such strategies are equitably feasible, acceptable, and impactful across a diverse range of settings and sub-groups (e.g., with varying levels of resources and structural impediments to health). This includes prioritizing collection of data on sustainment as an outcome using validated measures when possible (44), and assessing which strategies are particularly impactful in enhancing not just initial implementation efforts but long-term sustainability of EBIs (43). Existing tools like The *Acceptability, Practicability, Effectiveness, Affordability, Side-effects, and Equity* (APEASE) criteria developed by Michie et al. (45) may be useful for considering factors that impact the appropriateness of a strategy for a specific setting or context, and can inform the selection or co-design of strategies. Additionally, there is value in moving away from “one-time” implementation strategies implemented by external facilitators and toward greater focus on advancing understanding of strategies that are well-aligned with existing resources and expertise in practice settings, led by internal staff/practitioners, and that have the potential to build more durable community and organizational capacity (46, 47).

Tools like the Program Sustainability Assessment Tool and the Clinical Sustainability Assessment Tool may be useful in identifying key areas that partners perceive as critical for building more long-term capacity for sustainability and in informing the development and testing of sustainability strategies (43, 48, 49). Additionally, attention to more equity or context-specific frameworks that seek to understand sustainability determinants may be important in settings and populations experiencing inequities. Sustainability-specific frameworks like The Integrated Sustainability Framework can be refined or adapted for specific settings to attend to equity issues (6), informed by existing qualitative research guides that can inform this process (15). As one example, the Lay Health Advisor (LHA) Sustainability Framework was specifically developed in the context of LHA programs in African American communities and considers factors like mistrust and discrimination in shaping sustainability (14, 50). Health equity tools, including frameworks such as the PEN-3 cultural model that help build capacity for sustainability are complementary and can also be incorporated in contextual assessments to understand sustainability (38–41). As noted, such frameworks would allow a framing that moves away from a deficit mindset to one that is asset-driven about what communities can do to achieve the sustained use of EBIs to reduce health inequities. Frameworks like the PEN-3 cultural model offer potential to shepherd in new pathways of knowing, including increasing understanding of the complexity of factors that shape health inequities that continue to persist, but from a lens that is positive yet transparent about challenges and resources that matter in efforts to sustain the EBI (51).

#### Focus on equity and engagement in the context of both research and practice efforts

Prioritizing a focus on equity and engagement early and often along the translational continuum is essential, as it is the foundation of later sustainability. This requires fundamental shifts in how we approach, prioritize, and fund community-engaged implementation science research and the extent to which community-aligned and practice-based evidence is valued in our scientific paradigm (2, 52, 53). Community-engaged approaches have the potential to enhance and build capacity for sustainability and health equity by shifting more power, funding, and resources to value and support community partner time, evidence, and expertise (54). Making progress will also require that researchers consider developing more flexible and agile EBIs from the start that recognize the evolving nature of community and population needs over time, as well as the changing sociopolitical landscapes that can thwart sustainability efforts. While there has been progress on requiring a focus on equity and community engagement in recent grant announcements [e.g., (55)], there is a need for more grants and funding mechanisms that support community-led initiatives, facilitate resource sharing, and require more equitable decision-making/leadership between academic and community partners.

The foundation of sustained intervention delivery and impact is long-term partnership, which is essential to building the trustworthiness of researchers and institutions. This will necessitate more equitable decision-making and resources with community partners in the context of research, as well as institutional commitment and accountability to community partners beyond research grants from universities and healthcare systems. There is value in building and supporting infrastructure and processes at institutions to meaningfully engage and empower communities beyond short-term and unstable funding and grant cycles (56). A focus on sustainability and health equity requires transparency and bi-directional communication in identifying and achieving short and long-term benefits for both partners (e.g., identifying priority areas for long-term capacity-building that is valued by partners). Finally, it necessitates accountability of researchers and institutions to committing to action (regardless of grant outcomes and timelines) and collecting and returning data that is timely, accessible, meaningful, aligned with partner priorities, and is actionable in creating change (29). Such shifts in how research is conducted will require that institutions and funders place greater prioritization and resources toward supporting impactful partner-engaged research and dissemination of findings, and that there is greater value and recognition in academic promotion for community-engaged and equity-focused research.

In conclusion, as we have argued here, maximizing the population health impact of EBIs and addressing the research-to-practice gap requires prioritizing, investing, and proactively planning for the sustainability of EBIs (37), particularly in settings and populations experiencing health inequities. We have highlighted key gaps in the field and recommendations for future implementation science researchers and practitioners to advance the science and impact of work at the intersection of sustainability and health equity. We believe that implementation science is at an important crossroads with respect to how it can be applied and advanced to make progress toward health equity. We also note it is important to address health equity with efforts to sustain EBIs, keeping in mind the distribution of resources, power, and structural determinants of health equity over time within and across populations under-represented in research. Going forward, a resolute focus on fairness and justice with investments made in settings with limited resources requires additional insight into the long-term return of investment of research, including who benefits and who does not, the role of power, and the shared frustration that researchers and communities experience when EBIs end, despite promising findings (3, 57–59). Understanding the toll of health inequities and progress toward their eradication will be futile, unless equal efforts are made to sustain and continually improve EBIs that address these inequities. Only then will the promise of creating lasting conditions from the beginning, in which all can truly thrive, be realized.

## Data availability statement

The original contributions presented in the study are included in the article/supplementary material, further inquiries can be directed to the corresponding author.

## Author contributions

RS, MH, and JI made substantial contributions to the conception and design of the work and to the acquisition, analysis, or interpretation of data/literature for the work as well as substantial contributions to drafting the work and revising it critically for important intellectual content and provided approval for publication of the content and agreed to be accountable for all aspects of the work in ensuring that questions related to the accuracy or integrity of any part of the work are appropriately investigated and resolved. All authors contributed to the article and approved the submitted version.

## Conflict of interest

The authors declare that the research was conducted in the absence of any commercial or financial relationships that could be construed as a potential conflict of interest.

## Publisher’s note

All claims expressed in this article are solely those of the authors and do not necessarily represent those of their affiliated organizations, or those of the publisher, the editors and the reviewers. Any product that may be evaluated in this article, or claim that may be made by its manufacturer, is not guaranteed or endorsed by the publisher.
